# Quality Improvement of Pediatric Airway Emergency Carts: Standardization, Streamlining, and Simulation

**DOI:** 10.7759/cureus.39727

**Published:** 2023-05-30

**Authors:** Zachary J Fleishhacker, Douglas M Bennion, Jose Manaligod, Deborah Kacmarynski, Bonita Y Ropp, Sohit Kanotra

**Affiliations:** 1 Otolaryngology - Head and Neck Surgery, University of Iowa Hospitals and Clinics, Iowa City, USA; 2 Nursing, University of Iowa Hospitals and Clinics, Iowa City, USA

**Keywords:** simulation training, pediatric airway emergency, pediatric otolaryngology, quality improvement, airway cart

## Abstract

Objective

Pediatric airway emergencies are amongst the most tenuous scenarios faced by on-call providers, requiring quick access to the appropriate equipment and a timely response. In the present study, we report on the testing and improvement of pediatric airway carts at our institution. The primary objective was to optimize our pediatric airway emergency carts to improve response times. Secondarily, we aimed to implement a training scenario to improve providers’ familiarity and confidence in attaining and assembling equipment.

Methods

Surveys of airway cart configuration at our hospital and others were used to identify differences. Volunteer otolaryngology physicians were tasked with responding to a mock scenario using an existing cart or one modified based on the survey. Outcomes included (1) time to arrival of the provider with the appropriate equipment, (2) time from arrival to complete assembly of equipment, and (3) time for re-assembly of the equipment.

Results

The survey revealed differences in cart equipment and location. The inclusion of a flexible bronchoscope and a video tower, as well as the placement of the carts directly within the ICU, resulted in improved time to arrival by an average of 181 seconds, and improved equipment assembly time by an average of 85 seconds.

Discussion

Standardization of pediatric airway equipment on the cart and location near critically ill patients improved response efficiency. Simulation led to improved confidence and reduced reaction time among providers at all levels of experience.

Conclusion

The present study provides an example for the optimization of airway carts, which can be adapted by healthcare systems to their local milieu.

## Introduction

Among the clinical scenarios faced by the on-call pediatric intensivist, anesthesiologist, or otolaryngologist, pediatric airway emergencies rank highly with respect to both urgency and the potential impact on the overall outcome for the patient. When pediatric airway emergencies arise, a critical factor in the successful and timely securing of the airway is the immediate availability of the necessary equipment. Given the nature of pediatric airway emergencies, most institutions place a high priority on assembling and maintaining emergency pediatric airway carts.

Limitations of emergency airway carts that may potentially lead to a poor response include the lack of, or too few such carts or their inaccessibility, an absence of up-to-date or appropriately sized equipment, and insufficient familiarity of team members with their locations and contents. The readiness of otolaryngology-specific equipment carts improves patient care, helps contain costs, and, importantly, improves access to the most essential pieces of equipment for emergencies [1]. The introduction of video laryngoscopy for endotracheal intubation is a welcome advance in the care of pediatric patients with the ability to improve glottic visualization, the potential for higher intubation success, and the use of video recordings for education and quality improvement afterward [2]. Intubating video laryngoscopy systems are not the best resource in all situations, especially considering the equivocal data on the improvement of first-pass success rates and tracheal intubation-associated events [3,4]. Identification of patients who would be best suited for the use of video intubating technology is difficult given that bedside screening tests to identify pediatric patients with difficult airways do not exist [5]. It has been noted that difficult intubations are more common in neonates less than 32 weeks old and less than 1500 g in weight; this group has five times higher odds of adverse events (20-40%) and four times higher odds of severe oxygen desaturation [5]. Other clinical assessments and standard examination techniques have proven quite limited in screening patients with difficult airways. Further, a subset of patients present with scenarios wherein the airway can be visualized indirectly with intubating video laryngoscopy but for whom instrumentation and intubation are not possible due to extreme anatomic limitations (e.g., infants with Pierre Robin sequence). Readily available alternatives that employ indirect (e.g., flexible laryngoscopy or intubating video laryngoscopy) or direct (e.g., direct Parsons/Storz laryngoscopes) visualization of the airway continue to play a critical role. The integration of these tools with video technology has the advantage of broadcasting the view for all team members to see.

The experience at our institution has been that pediatric emergency airway scenarios occur most frequently in the ICUs. They also occur in the emergency room, but the previous literature has demonstrated that the stabilization of patients in this context is more effective, leading us to focus on the ICU [6]. At this institution’s pediatric ICU, airway emergencies are responded to by a multi-disciplinary team, including pediatric critical care physicians, pediatric anesthesiologists, and in-house otolaryngologists. While basic pediatric intubation equipment, including direct laryngoscopes and endotracheal tubes, are available in multiple locations within the ICU, difficult airway equipment is currently only quickly available on two airway carts, one located two floors above the ICU in the children’s OR and another located in the connected main hospital’s OR.

The patient safety/quality improvement (PS/QI) project described here was carried out to guide updates and improvements to this institution’s pediatric emergency airway carts, as well as to develop a rationale for standardizing equipment based on the available literature and an inter-institutional comparison. A simulation exercise was performed to assess the effectiveness of selected changes in a real-world setting. Our primary objective was to measure and improve provider response times to pediatric emergency airway scenarios in the pediatric intensive care unit through the implementation of studied changes to the emergency airway cart. Our secondary objective was to implement a training scenario to familiarize team members with the location and assembly of relevant equipment required for an emergency airway situation.

## Materials and methods

**Figure 1:**
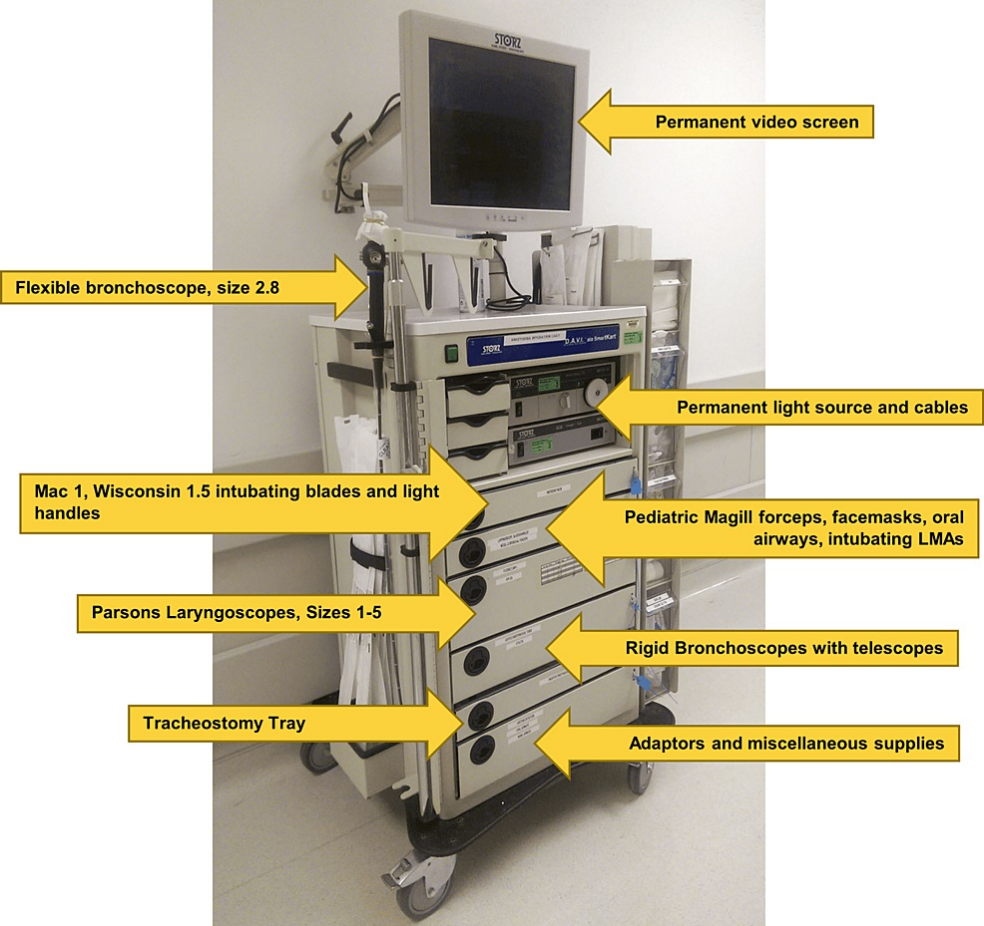
Suggested configuration of pediatric emergency airway cart. Includes addition of an onboard video tower, Macintosh 1 direct laryngoscope, a flexible bronchoscope, a needle cricothyrotomy kit, and pediatric sizes of equipment already present on existing carts LMA: laryngeal mask airway.

This PS/QI project was deemed exempt from IRB review (IRB#202110527). This PS/QI project and manuscript adhere to the Standards for Quality Improvement Reporting Excellence (SQUIRE 2.0) guidelines [7]. This project was divided into two phases. The first phase consisted of the administration and analysis of an informal inter-institutional survey of three large academic pediatric hospitals, including our own, as outlined in Table 1. The second phase was an intervention involving this institution’s current pediatric airway carts and subsequent measurement of outcomes in simulated emergencies. Responses to the survey and review of the current literature were used to guide the design of a standardized, “ideal,” pediatric airway emergency cart (Figure 1).

**Table 1:** Inter-institutional comparison of pediatric emergency airway carts conducted by a survey

Equipment	Home institution	Pediatric hospital #1	Pediatric hospital #2
Ped airway cart	One per OR area	One per ICU	One total
Storz video tower	One in the otolaryngology clinic	One per cart	None
Lindholm scopes	Infant, toddler, child	None	None
Parsons scopes	Sizes 1-5	Sizes 1-5	Sizes 1-5
Other scopes	None	Benjamin Lindholm, Pierre Robin, Mac 1	None
Rigid bronchoscope, size 2.5 to 3.5	Sizes 2.5, 3, 3.5	Sizes 2.5, 3, 3.5, multiple lengths	Sizes 2.5, 3, 3.5
Rigid bronchoscope, size 3.7 to 6	None	Sizes 4, 5, 6, multiple lengths	Sizes 3.7, 4, 5, 6
Telescopes	Sizes 2.8, 4	Sizes 1.9, 2.8, 2.9, 4, 5	Sizes 1.9, 2.8, 4, multiple lengths
Flexible bronchoscope	None	Sizes 2.8, 5.2	None
Needle cricothyrotomy kit	None	Yes	None

**Figure 2:**
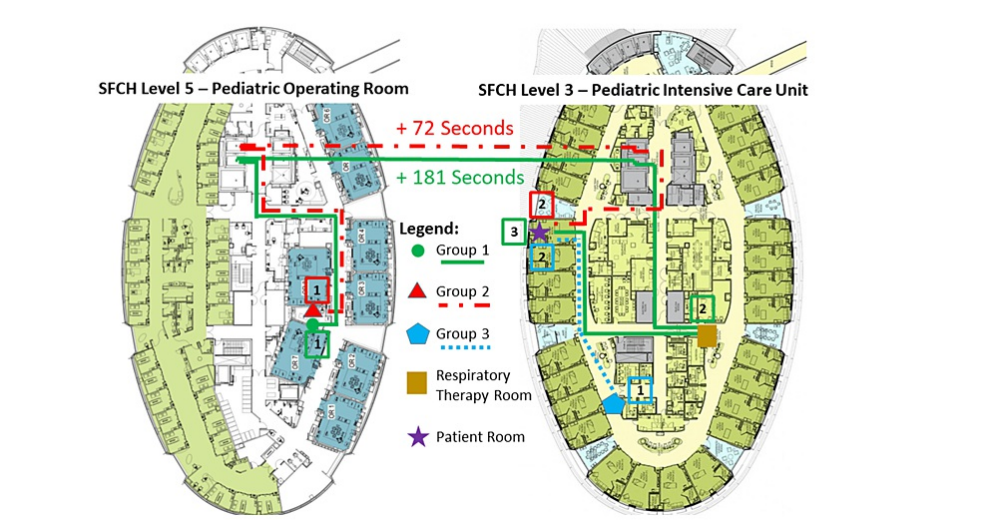
Map of our institution’s current emergency airway cart route and proposed improvements by simulation group SFCH: Stead Family Children's Hospital.

The effectiveness of this planned intervention was assessed in a mock pediatric emergency airway scenario. Volunteer physicians from the Department of Otolaryngology were randomly divided into simulation groups and given a four-hour time window in which a page would be sent indicating that the mock scenario had begun in a predetermined, empty, pediatric ICU room. Participants included resident physicians who take in-house calls covering the pediatric ICU, a pediatric otolaryngology fellow, and pediatric otolaryngology faculty. The participants responded as if it were a true emergency, gathering all necessary equipment using either the old airway carts or their updated counterparts (see Figure 2 below and its description in the results section). All participants began the exercise in the same location (on-call resident physician work room) and responded to a simulated emergency in the same room within the pediatric ICU. Upon arrival at an unoccupied ICU room, they were asked to assemble the following pieces of equipment as quickly and completely as possible: (1) a direct laryngoscope with a working light source attached; (2) a rigid bronchoscope attached to a video monitor and light source; and (3) a flexible bronchoscope with suction tubing, light source, and video monitor attached. The task was deemed complete when all equipment was functionally assembled, including a light source and video display. After this task was completed, the equipment was disassembled and participants were briefly instructed on proper assembly. Participants then repeated the timed assembly task. Additionally, both prior to the simulation and after the second attempt at assembly, the participants were asked to complete a survey assessing knowledge and comfort with the institution’s current pediatric emergency airway cart arrangement (site of storage and configuration). Each participant went through the simulation only once. All simulations, data collection, and surveys were conducted at the same institution over a two-day period in February 2022.

The primary outcome measured was the time from the mock page being sent to the delivery of equipment to the room. Secondary outcomes were times of equipment assembly on both first and second attempts and responses on the pre- and post-intervention surveys. Data collection tools included a stopwatch for all timing purposes and a web-based survey form administered both before and after participation in the simulation.

Statistical analysis was performed using GraphPad Prism software (GraphPad Software, San Diego, CA). The primary outcome, i.e., time to delivery, was analyzed using both a one-way ANOVA and an unpaired single-tailed Student's t-test for pairwise comparisons. The secondary outcomes (i.e., times for first and second attempts at equipment assembly and pre- and post-survey data) were compared using an unpaired, two-tailed Student's t-test.

Neither patients nor patient information were utilized in this project. Also, participants were instructed to not respond to the mock page if involved in urgent patient care. All relevant teams (pediatric ICU, otolaryngology, and anesthesia teams) were aware of the times of data collection and locations of the emergency airway carts in case a true airway emergency arose. An unoccupied pediatric ICU room was utilized with the unit director's approval.

## Results

Our inter-institutional survey of three stand-alone pediatric hospitals included our own 190-bed hospital, a 400+-bed hospital, and a 250+-bed hospital. This survey assessed each institution's pediatric emergency airway cart and was used as displayed in Table 1. Commonalities included the presence of Parsons scopes and certain sizes of rigid telescopes. For our institution’s airway cart, we noted absences of the following compared to similar institutions: a video tower, a flexible bronchoscope, and pediatric sizes of various types of equipment already present (e.g., oral airways and rigid telescopes). An additional difference was the location of the cart. The survey revealed that in addition to a cart located in the operating room (OR), an ideal arrangement would include updated airway carts in the neonatal and pediatric intensive care units (PICUs).

**Figure 3:**
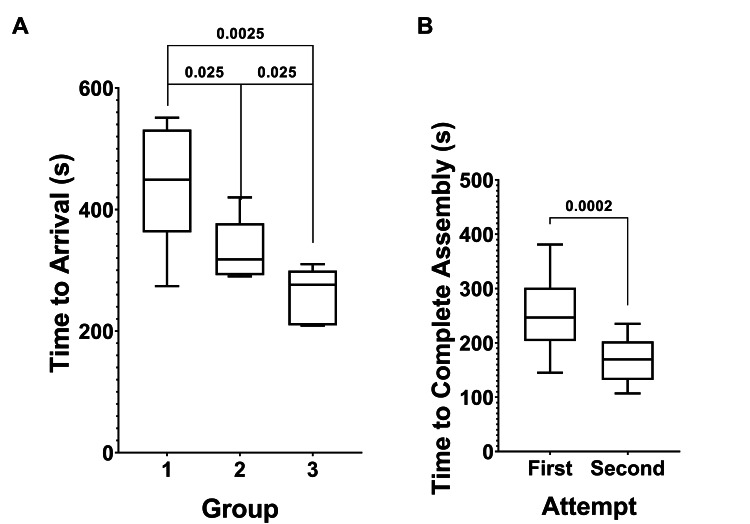
Response and equipment assembly times (A) Response times to a mock pediatric emergency airway in the pediatric intensive care unit (PICU) by simulation group. (B) Time to complete and correct assembly equipment on the first attempt and subsequent immediate second attempt. P-values are displayed above graph bars. P < 0.05 is considered statistically significant.

For simulations of the airway carts in practice, 16 participants were enrolled and randomly divided into three groups, as illustrated in Figure 2. Group 1 (n = 6) collected equipment from two sites: the OR area (two levels above the PICU), which currently houses the pediatric emergency airway cart; and the PICU respiratory therapy room, where a video tower and flexible bronchoscope are stored. Group 2 (n = 5) collected equipment from a single site, the OR area, obtaining both the airway cart (as was the case for Group 1) and the video tower and flexible bronchoscope from this location. Group 3 (n = 5) also collected equipment from a single site; in this case, it was the PICU, which housed an updated airway cart that included a video tower and flexible bronchoscope. The mean time to arrive with the emergency airway cart, video tower, and flexible bronchoscope was significantly shorter for Group 3 than for the others (p = 0.0041, Figure 3A). Mean times to arrival were 440 seconds for Group 1, 331 seconds for Group 2, and 259 seconds for Group 3. The mean times for the first and second attempts at equipment assembly were 253 seconds and 168 seconds (p = 3.5 x 10-4, Figure 3B).

Participants were surveyed about their familiarity with and confidence working with the equipment during the simulated airway emergency, and data are presented in Table 2. Notable changes were a significant increase in knowledge of the location of airway cart equipment (4.23 +/- 0.83 vs. 2.64 +/- 0.83, p < 0.005), comfort with the assembly of equipment (4.38 +/- 0.65 vs. 2.29 +/- 0.83, p < 0.005), and confidence in the ability to respond quickly to a page for a pediatric airway emergency (4.00 +/- 0.82 vs. 2.36 +/- 1.15, p < 0.005). Participants also reported being dissatisfied with the current location of the pediatric emergency airway cart (2.00 +/- 1.15).

**Table 2:** Participants' responses to the pre- and post-intervention survey Scores ranged from 1 to 5, with 1 indicating strong disagreement and 5 indicating strong agreement with the prompt.

Survey prompt	Pre-intervention, mean (+/- SD)	Post-intervention, mean (+/- SD)	p
I have adequate knowledge of the current location of pediatric emergency airway cart equipment.	2.64 +/- 0.83	4.23 +/- 0.83	0.00025
I am comfortable with the assembly of pediatric emergency airway equipment (bronchoscope, direct laryngoscope, video tower).	2.29 +/- 0.83	4.38 +/- 0.65	1.20E-07
I am confident in my ability to respond to an emergency airway page with appropriate equipment in a timely manner.	2.36 +/- 1.15	4.00 +/- 0.82	0.00026
The current emergency pediatric airway cart is located in an ideal place where urgent pediatric airway situations may arise.	2.21 +/- 0.89	--	--
The current emergency pediatric airway cart has all the right equipment for an emergency airway scenario onboard.	2.93 +/- 0.73	--	--

## Discussion

The results of this multi-phase quality improvement project include the identification of inter-institutional differences in airway cart configuration and avenues for improving this institution’s pediatric emergency airway carts. Addition of a flexible bronchoscope and video tower to the carts, as well as placement of the carts in the PICU, significantly improved provider response times by more than three minutes in a situation where every second counts. Significant improvement was seen when the equipment was consolidated, even in the absence of a change in location (109 seconds); with the additional change in the location of the cart, there was a further shortening of the time (72 seconds). Improvements were also seen as participant familiarity with the pediatric airway cart equipment increased; assembly times improved by nearly 1.5 minutes on the second attempt, as did confidence in responding to a page for a potential airway emergency. Collectively, our interventions led to a nearly five-minute improvement in the response during a simulated pediatric airway emergency. Our results are consistent with previous successful attempts to improve airway cart efficiency by consolidating equipment at a single location and removing infrequently utilized equipment; this led to a 77% reduction in response time and a 74% reduction in distance traveled during the emergency airway response [8].

The topic of this study is important because the pediatric population is especially vulnerable to severe hypoxia during the compromise of the airway because of their anatomy, increased metabolic oxygen consumption, and low cardiopulmonary reserves [9]. The sequelae of prolonged hypoxia due to a delayed response to a pediatric airway emergency include risk of death, hypoxic-ischemic encephalopathy, and long-term damage to vital organs (with each second of inadequate oxygenation [10,11]. Optimization of the procedures and reduction of response times in these scenarios is critical to preventing these devastating adverse outcomes. Such streamlining is of heightened relevance for urgent airway consults at institutions like ours where such situations arise only intermittently, averaging one to two per month.

The pediatric airway emergency response is multidisciplinary, requiring interactions and communication among multiple teams [12]. The use of a flexible bronchoscope represents a useful alternative to a direct laryngoscope, an instrument used more commonly by otolaryngologists. The inclusion of the video bronchoscope allows providers without this specialized training to effectively utilize the airway emergency cart [13]. For example, in a single-center retrospective study of 66 bronchoscopies performed on newborns using a flexible bronchoscope, six were done for emergency tracheal intubation and all were successful [14]. Thus, intubation using a flexible bronchoscope is a safe and effective method for securing the airway in an emergency setting [14]. Furthermore, in certain clinical scenarios (e.g., severe Pierre Robin sequence or type III laryngeal cleft), the additional length of the bronchoscope would make it possible to verify that the endotracheal tube is placed at the appropriate depth. One report listed several additional items as being valuable. These included a curved intubation blade, Magill forceps, a pediatric face mask, intubating laryngeal mask airway (LMA), and oral airways [15]. Additionally, the inclusion of a tracheostomy equipment tray provides the provider with a definitive surgical management option for when all other adjuncts prove unsuccessful. Our equipment recommendations are consistent with those of the national organizations of other countries for providing streamlined access to airway equipment for at-risk patients in ICUs [16].

Of note, some institutions have adopted a policy of packaging airway instruments stored on emergency carts in a sterile manner to decrease infection risk. A recent retrospective chart review compared infection rates and time to assemble instruments for two groups of 100 patients, one before and the other after such a policy was implemented for otolaryngology airway carts at a single institution. This revealed that neither the infection rate nor length of hospital stay decreased and that the time required for equipment assembly doubled [17]. Another study reported that reducing the amount of packaging on the emergency airway cart reduced both assembly time and time to visualization of the trachea by two to four minutes [18]. Both the original and redesigned carts in this study contained individually packaged, Joint Commission-compliant equipment sets, including a Parsons laryngoscope, rigid bronchoscope, camera, and various adaptors.

According to our survey, participants in the simulation exercise experienced an increase in knowledge, comfort, and confidence in dealing with pediatric emergency airway scenarios. This is consistent with previous demonstrations of the efficacy of simulation training across disciplines, in areas including emergency medicine [19,20], otolaryngology [21], and anesthesia [12,22]. Our finding that the response to an airway emergency is improved by familiarity with the equipment itself is also consistent with other studies showing that providers are most confident with individual pieces of equipment that are used in practice more frequently [23,24], and when they have recently used an emergency equipment cart [25]. These data support periodic training for providers who are expected to respond to pediatric emergency airway situations infrequently.

The results of this PS/QI study support the consolidation of all potentially necessary equipment onto a single emergency pediatric airway cart, its placement in a location near likely sites of an airway emergency, such as in the OR and the PICU, and the inclusion of equipment that enables videoscopic intubation. The improvement in response times observed in our study can be attributed to multiple factors. One is that consolidation prevents providers from having to collect equipment from multiple locations, allowing a more rapid response to the location of the emergency. A second factor is that consolidation makes it easier for a team member other than the provider (such as a nurse or respiratory therapist) to bring all necessary equipment to the patient’s room while the provider responds, potentially further reducing the response time in a true emergency. A third factor is that appropriate placement of the airway cart reduces time spent traversing the hospital; as one participant observed, “having to take the elevator down to the PICU (from the OR) with the emergency cart is what took the longest.” A fourth factor is that not having a dedicated PICU airway cart can lead to delays that are harder to anticipate, such as the need to change scrubs or put on appropriate personal protective equipment (PPE) to enter the OR area or to have OR staff retrieve the airway cart.

This PS/QI project has several limitations. One is that the number of participants (n = 16) is small and limits the power of the statistical analysis, although the primary outcome is strongly supported by the difference in outcomes between Groups 1 and 3 (Figure 3A). However, the number of participants is strong in the context of single-site studies of otolaryngology trainees. Others are that the inter-institutional survey was limited by specificity to the type of hospital (stand-alone pediatric), the small number of sites, and the lack of formal assessment tools. Subsequent surveys of a more diverse array of inpatient pediatric otolaryngologic practice models would be beneficial. We also recognize that the impact of our proposed changes was not assessed in actual patient scenarios, and unknown factors inherent to each patient consultation are likely to contribute to variability in improvements in response times and outcomes. However, similar recommendations have been implemented in other clinical settings and resulted in an increase in the success of first-pass intubation attempts (by 29%), a reduction in the frequency of hypoxic adverse events (4/46 post-intervention vs. 10/71 pre-intervention), and a reduction in the number of hypotensive adverse events (0/46 post-intervention vs. 15/71 pre-intervention) [25]. Our study also did not assess other factors that might have contributed to a response delay, including the skills of individual providers with the equipment and their experience with its packaging. An additional consideration for future efforts to optimize emergency airway response time is a closer examination of the packaging of equipment and its organization on the cart.

## Conclusions

The results reported here illustrate a successful intervention and improvement in provider response times to pediatric airway emergencies and in their familiarity with the equipment needed during such responses. We expect that if all identified changes to carts and training were implemented, our response times in urgent scenarios would be reduced by nearly five minutes. Our findings provide a foundation for a recommendation to add a video tower and flexible bronchoscope to existing pediatric emergency airway carts when they are not already present, as well as to purchase an additional cart(s) with the same collection of equipment for placement in physically distant pediatric ICU areas. Additionally, these data support holding periodic training simulations for all providers who may have to respond to a pediatric emergency airway scenario.
